# Quantifying Human Avoidance Behavior in Immersive Virtual Reality

**DOI:** 10.3389/fnbeh.2020.569899

**Published:** 2020-09-30

**Authors:** Florian P. Binder, Victor I. Spoormaker

**Affiliations:** ^1^Department of Translational Research in Psychiatry, Max Planck Institute of Psychiatry, Munich, Germany; ^2^International Max Planck Research School – Translational Psychiatry, Max Planck Institute of Psychiatry, Munich, Germany

**Keywords:** anxiety, fear conditioning, avoidance, virtual reality, reinforcement, BAT, forced-choice, search task

## Abstract

Avoidance behavior is a key symptom of most anxiety disorders and a central readout in animal research. However, the quantification of real-life avoidance behavior in humans is typically restricted to clinical populations, who show actual avoidance of phobic objects. In experimental approaches for healthy participants, many avoidance tasks utilize button responses or a joystick navigation on the screen as indicators of avoidance behavior. To allow the ecologically valid assessment of avoidance behavior in healthy participants, we developed a new automated immersive Virtual Reality paradigm, where participants could freely navigate in virtual 3-dimensional, 360-degrees scenes by real naturalistic body movements. A differential fear conditioning procedure was followed by three newly developed behavioral tasks to assess participants’ avoidance behavior of the conditioned stimuli: an approach, a forced-choice, and a search task. They varied in instructions, degrees of freedom, and high or low task-related relevance of the stimuli. We initially examined the tasks in a quasi-experiment (*N* = 55), with four consecutive runs and various experimental adaptations. Here, although we observed avoidance behavior in all three tasks after additional reinforcement, we only detected fear-conditioned avoidance behavior in the behavioral forced-choice and search tasks. These findings were largely replicated in a confirmatory experiment (*N* = 72) with randomized group allocation, except that fear-conditioned avoidance behavior was only manifest in the behavioral search task. This supports the notion that the behavioral search task is sensitive to detect avoidance behavior after fear conditioning only, whereas the behavioral approach and forced-choice tasks are still able to detect “strong” avoidance behavior after fear conditioning and additional reinforcement.

## Introduction

Avoidance behavior is a key symptom of most anxiety disorders (American Psychiatric Associaton, 2013) and a central readout in animal research (Lonsdorf et al., 2017). There are numerous well-established tests to assess fear-related behavior in animals (Bailey and Crawley, 2008). In humans, the objective quantification of overt avoidance behavior is typically restricted to clinical populations. In the behavioral approach test (Grös and Antony, 2006), for example, individuals with a specific phobia have to approach the phobic stimulus whereby the distance to it functions as primary readout. Naturally, this test is only effective for intense fear, such as in phobia.

To measure more moderate fear in a healthy sample, other methods are required to quantify avoidance behavior. In laboratory settings, human avoidance behavior is currently assessed by questionnaires or computer-based tasks, during which button presses or a joystick navigation on the screen serve as measurement of behavior. This has provided valuable insights, for example, into the mechanisms of avoidance learning (Pittig et al., 2020), the effect of cost of avoidance (Rattel et al., 2017), or sex differences (Sheynin et al., 2014). Due to their experimental nature, avoidance tasks to date are primarily focused on avoiding the aversive event. However, anxiety disorders are characterized by the avoidance of the antecedent stimulus (i.e., the spider) and not necessarily the aversive event only (i.e., the bite of a spider). In order to reflect this characteristic, we need more experimental paradigms that investigate the avoidance of the antecedent stimulus (Krypotos et al., 2015). Furthermore, we need more ecologically valid and sophisticated designs that model ambiguity and conflict to fully understand the pathological mechanisms of avoidance in anxiety disorders and optimize treatment (Beckers et al., 2013; Pittig et al., 2018).

The recent technological development of immersive Virtual Reality (iVR) allows the objective tracking of human behavior with high precision in experimentally designed virtual contexts. These contexts are generated by a computer and presented to the participant in a sufficiently convincing manner to suspend disbelief and to become fully engaged with the context. Navigation is more natural as participants can walk around and grab objects intuitively. All motions can be recorded using sensors on the torso and limbs and can be extended with simultaneous subjective or physiological readouts, such as ratings or heartrate. Compared to experiments in real contexts, experiments in iVR can be fully automated yielding a high level of standardization. Finally, participants can be easily transferred from one context to another. The potential of this technology has been shown, for example, in the study of Biedermann et al. (2017). They translated the elevated plus-maze to iVR, in which participants walked on a wooden plus-shaped maze with two closed arms being surrounded by rocks and two open arms being in the air. They observed that participants with high trait anxiety spent less time walking on the open arms than participants with low trait anxiety. Studying behavior with such an integrated set-up could help us translate preclinical findings to humans and expand our understanding of human avoidance behavior. Ultimately, the quantification of avoidance behavior might be beneficial for monitoring the progress of exposure treatment in patients.

The question is, how can avoidance behavior be experimentally induced in healthy participants? In animal research, a well-established model to induce fear-behavior is fear conditioning (Milad and Quirk, 2012; LeDoux et al., 2017). It entails the repeated pairing of a neutral stimulus with an intrinsically aversive event, such as a mild electrical shock. The former neutral stimulus is called the conditioned stimulus (CS+) and the aversive event is the unconditioned stimulus (US). In a differential Pavlovian conditioning paradigm another stimulus is added, which is never followed by the US, resulting in a safety stimulus (CS−). In line with animal work, previous work in humans has revealed that approach/avoidance tendencies manifest after fear conditioning in computer-based tasks, with a joystick or button press (Cornwell et al., 2013; Krypotos et al., 2014; Rattel et al., 2017).

Initial work has shown that fear conditioning is effective using iVR (Kroes et al., 2017). This study used a procedure where participants were sitting on a chair and were automatically navigated on a predefined path through virtual rooms. This was necessary to exclude idiosyncratic behavior during conditioning. They observed reliable acquisition of subjective fear (arousal and valence), physiological fear responses (electromyography startle responses, and skin conductance responses) and showed iVR to be an effective tool to investigate human contextual processes. This study raises the question of how participants would behave in such a context, if they had more degrees of freedom or if avoidance had been made less explicit, as this could affect the sensitivity of the tasks.

To investigate these questions, we developed a new procedure in iVR, in which differential fear conditioning was followed by three tasks to assess the behavior of participants towards the conditioned stimuli: a behavioral approach task with the aim to translate the behavioral approach task to healthy human participants by instructing them to touch the CS+ and CS-; a forced-choice task, in which participants chose between a path alongside the CS+ or a path alongside the CS-; a behavioral search task, in which participants could move freely within a squared area with the CSs presented on opposite sides, and a gaming component to induce movement. These three tasks allowed us to compare varying instructions, degrees of freedom and high or low task related relevance of the stimuli on the sensitivity of the task to detect avoidance behavior. Furthermore, the manipulation of the order of the behavioral tasks enabled us to investigate the effect of additional reinforcement in previous tasks on avoidance behavior in the test task. In this article we present two experiments: An exploratory quasi-experiment to explore initial effects and a second confirmatory experiment with randomized group allocation to test the robustness of the effects.

## Quasi-Experiment

### Materials and Methods

#### Participants and Runs

A total of 60 healthy individuals participated in the four runs of the quasi-experiment. They were recruited through a variety of means including a notice at local universities and advertisements on the institute’s website and on social media. We excluded 5 participants: 1 participant misunderstood the instructions, 3 participants reported after the experiment that they had not learnt the CS-US contingencies, and 1 participant did not see the balloons during the behavioral tasks. A total of 55 participants (*M* = 24.3, *SD* = 4.2, range: 18–34, female: 30) were included in the analyses. The measurements took place in the afternoon between noon and 6 p.m. The study protocol was approved by a local ethics commission (Faculty of Medicine at Ludwig Maximilian University of Munich; project number: 18–403) and was conducted in accordance with the Declaration of Helsinki (2013).

The quasi-experiment consisted of four different runs. In each run, a group of participants underwent the experiment with the same protocol. Afterward, a few manipulations on the protocol were made for the next run. See Supplementary Table 1 for a detailed description of all manipulations.

#### Setup

The VR was generated in Unity 3D Pro (version 2018.3) with a sampling rate of 90 frames per second. We used the HTC Vive with controllers and in-ear headphone to present the VR, which was connected to Steam VR.

Participants were free to move around the laboratory (room of 4.6 m × 5.5 m), which spatially agreed with the virtual scenes: three sides were aligned with the respective wall; one side was shortened because of the desk with the desktop computer, resulting in a field of 4.6 m × 4.3 m. In order to increase the participant’s sense of presence, we deactivated the chaperone, which is a safety grid in the virtual environment indicating the border of the field. Instead, the borders of the field were indicated as walls, wood blanks, or cordons. The cable of the HTC Vive was held by a trained person to ensure participants could move freely.

Electrocardiography was measured with the one channel eMotion Faros 180 device from BioSign. It was connected via Bluetooth to the computer, operated in online mode, and recorded with 250 samples per second. The Faros device was synchronized with Unity at startup. From this point on, package numbers of the received data were used to determine the time of the signal. This ensured that communication delays, due to buffering in the Bluetooth connection, did not affect data quality.

The body motion data was recorded with the Perception Neuron V2 System using 18 sensors on the torso, limbs, and head. It was wirelessly transferred to the Axis Neuron software (version 3.8.42.6503), where the accelerations of the sensors were converted in directed positions of 25 human body parts. The Perception Neuron Unity-Plugin (version 0.2.11) received these positions and used them to animate the default Perception Neuron avatar, which represents the body of the participant. We ignored the position in the room from the motion tracking system and instead used the precise position of the head-mounted display (HMD), to which we fixed the head of the avatar. With that we eliminated the global drift, which is a well-known error in inertial motion tracking systems, induced by the many summations of the acceleration over time (Lopez-Nava and Munoz-Melendez, 2016). The size of the avatar was adjusted to fit the body size of the participant. All motions were recorded in Unity by saving the global position and rotation of all 25 body parts of the avatar in every frame.

We used the PsychLab SHK1 constant current shocker (60 Hz AC) for 100 ms-duration electrical shocks (0.8–5 mA), as performed by Schmitz and Grillon (2012). It was connected to the computer via USB and was controlled directly from Unity. The electrode cable was extended by a 10 m custom produced cable of the manufacturer. The two electrodes were mounted to a piece of leather to fix the center distance to 2 cm.

#### Procedure

In the announcements, interested participants were asked to send an e-mail to apply for participation. The response of this mail contained a link to an online questionnaire covering the inclusion/exclusion criteria. Eligible participants were immediately redirected to a webpage on which they could choose their preferred timeslot of participation. One day before the experiment, they received a reminder of their appointment including a link to an online questionnaire that had to be filled out before the experiment. It consisted of Trait Anxiety (Spielberger, 1983), Big Five Inventory (Rammstedt and Danner, 2017), Intolerance of Uncertainty (Gerlach et al., 2008), Short Resilience Scale (Leppert et al., 2008), Beck-Depression Inventory II (Kühner et al., 2007), Motion Sickness Susceptibility Questionnaire revised (Golding, 1998), Anxiety Sensitivity Index 3 (Kemper et al., 2009), Sensation Seeking Scales, Form V (Beauducel et al., 2003), and the CID-Screener (Wittchen et al., 1999).

When participants arrived in the laboratory, they were informed about the procedure and gave their written informed consent. The two electrodes (55 mm; Ag/AgCl pre-gelled) for the electrocardiography were attached under the right collarbone and on the lower left ribs. The electrodes for the electrical shocks were attached to the left calf with an elastic bandage. The motion tracking sensors were placed and calibrated according to sex and body size, following the guidelines in Axis Neuron. The participant put on the head mounted display and the experiment started.

After the experiment, all sensors were detached and participants received a tablet device on which they rated their general anxiety in VR (VAS), US intensities, CS valences, evaluation of the duration in VR, nervousness at the beginning of the experiment, and filled out a few additional questionnaires: the Simulator Sickness Questionnaire (Kennedy et al., 1993), and the Presence Questionnaire Version 3 (Witmer et al., 2005).

#### Virtual Scenes

The first scene was the Tutorial, which was already loaded before the participant put on the HTC Vive. After the start of the experiment all (pre-recorded) instructions ran automatically. The Tutorial was followed by the Fear Conditioning task and the experiment continued with the three behavioral tasks in a counter-balanced but predefined task sequence. The experiment ended with the Recall task.

##### Tutorial

The Tutorial was a room, like the laboratory. It served to habituate participants to VR and to familiarize them with the VR-interaction. At the beginning, they received the instruction that they can move around as they would in the real world and should not walk through virtual objects or walls, as these could be covered by real ones. The controller was explained, and its handling rehearsed. The participant walked through the whole room once. The collection, carrying and dropping of objects was explained and trained. The shock intensity was also calibrated (Schmitz and Grillon, 2012). The occurrence of a shock during calibration was indicated by a 2 s countdown on a monitor on the wall. The intensity of the first shock was zero (no shock) in order to familiarize them with the procedure before the first real shock occurred. Afterward participants were asked to rate the intensity of the shock on a 5-point Likert scale (anchored with 1 = hardly noticeable, 2 = noticeable, 3 = unpleasant, 4 = very unpleasant, 5 extremely unpleasant). If they rated 1–3 the intensity was increased by one step of 0.82 mA and another shock was presented. This increment was chosen as it reached the maximum 5 mA with six equally sized small steps. If the participant rated the intensity as 5, the shock was decreased by a third of a step (0.27 mA). When they rated the shock as 4 the calibration was over, and this intensity was used for the rest of the experiment.

##### Fear Conditioning

**FIGURE 1:**
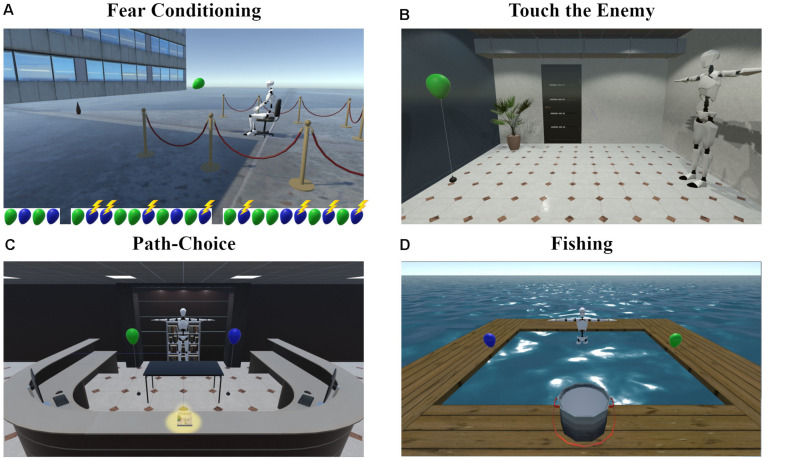
Screenshots of the virtual scenes for **(A)** Fear Conditioning and Recall, **(B)** Touch the Enemy, **(C)** Path-Choice, and **(D)** Fishing. The balloon sequence in **(A)** represents the trial order during habituation and both fear acquisition blocks with green representing the CS− and blue the CS+. Reinforced trials are marked with a flash. Note that the 2D screenshots do not convey the 3D-360 degree view that participants encountered in iVR. The screenshots are depicted from an allocentric standpoint for illustration purposes only. Participants experienced all scenes in the first-person view and were always able to move freely by naturalistic body movements.

During the Fear Conditioning task (see Figure 1A) participants sat on a chair in an open square. The CSs were green and blue balloons, which were inflated out of a vase, 6 m in front of the participant. They floated for 8 s toward the participants and then passed them by. The inter-trial-interval was set to 9 s. One of the balloons (CS+) was followed by the US after 6 s in 80% of trials, when the balloon was in closest proximity to the participant. The other colored balloon (CS-) was never paired with the US. The CS-type to balloon-color relation was counter-balanced between participants. Participants were instructed that this is a learning task, in which various balloons are shown and unpleasant stimuli can occur and that their job is to find out what will happen and when. In a short habituation phase, both balloons were presented two times each, without US. Afterward, a large monitor emerged from the ground and each CS was presented together with the question for the rating: “How likely does an electrical shock occur?” Participants rated their US probability on a Likert scale with eleven steps from 0 to 100%. After the habituation phase, participants were informed that from now on unpleasant stimuli can occur and at the very first rating they should simply guess. The subsequent fear acquisition contained two blocks in which both CSs were presented five times each, in a pseudorandom order. Between these blocks, as well as at the beginning and at the end of these blocks, ratings took place. After the acquisition phase participants were asked to stand up. They received the instruction that in all following tasks the unpleasant stimuli may occur again, before the next worlds were loaded.

##### Behavioral Approach Task (“Touch the Enemy”)

In this task (see Figure 1B and Supplementary Video 1), participants were instructed to touch the floating CS on the other side of the room, which was tied to the floor and not moving. After a countdown period of 10 s, participants had to walk across the room and to touch the CS with their right hand. This was done for each CS twice, in alternating order. The type of CS presented first was counter-balanced between participants.

##### Behavioral Forced-Choice Task (“Path-Choice”)

In Path-Choice (see Figure 1C and Supplementary Video 2), the task was to collect a book from the counter on the opposite side of a reception area in a lobby and place it in the rack on the backside, where participants had started. A large table stood in the center of the room to force participants to pass it either on the left or on the right side. One of the CSs was presented on each side of the table, this way participants were forced to choose between passing the CS+ or CS- when crossing the room. There were five trials: After a book was put in the rack, the next one appeared. The first book was placed in the center of the counter with both paths having the same length. The other books were placed on the right (book 2), on the left (book 3), on the far right (book 4), and on the far left (book 5). The position of the CS+ (left or right) was counterbalanced between participants and the CSs swapped after book 1 and book 3 occurred.

##### Behavioral Search Task (“Fishing”)

Participants stood in hip deep, non-transparent water and were instructed to try to catch fishes with a hand-net (handle length: 0.75 m, net diameter: 0.40 m) in their right hand (see Figure 1D and Supplementary Video 3). The field was surrounded by a wooden walkway and participants were told to stay within it. The start position was in the middle of the long side, facing at the field. The two CS+ and CS− were floating in the wind and tied to the short left and right sides of the wooden walkway. The placements of the CSs were counterbalanced between participants. Participants were informed that they cannot see where the fish are, this way we kept them unaware of the absence of fish in the water. Lastly, after 2 min of fishing, regardless of the participant’s position, if the hand-net was in the water for more than 0.5 s, one fish was automatically placed in the net and the controller vibrated, indicating the success. Finally, participants were told to drop the fish in the pot on the walkway.

##### Recall

The Recall task was in the same context as the Fear Conditioning task (see Figure 1A), but differed from it in five aspects: (1) There was another explanation at the beginning, saying that the task is the same as before, only shorter. (2) There was no habituation phase. (3) Both types of CSs were presented four times each. (4) CS presentations were not reinforced anymore. (5) Ratings were only acquired at the beginning and at the end of the task.

#### Statistical Analyses

All analyses were performed in Matlab R2019b and figures were generated with the “Gramm” toolbox (Morel, 2018). The η^2^ for analyses of variance (ANOVA), the Glass’ Δ for two sample *t*-tests, Hedges’ g_1_ for one sample *t*-tests, and Cohen’s U3 for Mann-Whitney *U*-tests were calculated with the Matlab-toolbox “Measures of Effect Size” version 1.6.1 (Hentschke and Stüttgen, 2011). For repeated measure analyses of variance (rmANOVA), we calculated the partial-eta-squared ($\eta_{p}^{2}$) and generalized-eta-squared ($\eta_{G}^{2}$) (Olejnik and Algina, 2003; Bakeman, 2005) effect sizes.

#### Heart rate analyses

The PhysioNet-Cardiovascular-Signal-Toolbox (version 1.0.2; Poian et al., 2019) was used to detect R-peaks in the electrocardiography-signal. RR-Intervals were calculated as differences between successive peaks and related to the time of the second peak. The resulting RR-timeseries was linearly interpolated with a sampling rate of 250 samples per second and all values higher than 1.5 s were marked as missing.

##### Task specific grouping: temporal-position and CS+- Experience

The *temporal-position* is a task specific partitioning of participants based on the individual position of the task in the task sequence. The temporal-position one, two, and three contain all participants who had the specific task as first, second, or last behavioral task, respectively.

To investigate the effect of additional reinforcement on avoidance behavior, we defined the *CS*+-*Experience* as categorization of possible manipulations of the CS-US contingency after the Fear Conditioning task, but before the respective task. Participants from temporal-position two or three were assigned to exactly one of these mutually exclusive categories: *no-approach* means the participant had the chance, but never approached the CS+ in any preceding task; *reinforcement* means the participant approached the CS+ at least once and every approach was reinforced; *non-reinforcement* means the participant approached the CS+ at least once and the approach was never reinforced; *mixed-reinforcement* is a mix of reinforcement and non-reinforcement and means the participant approached the CS+ at least twice, where at least one approach was reinforced and at least one was not. This categorization was also task specific: For instance, a participant with the order Touch the Enemy, Fishing, and Path-Choice could be in the category reinforcement for the Fishing task, but in the category mixed-reinforcement for the Path-Choice task, if there was an unreinforced approach during the Fishing task. Effects of the CS+-Experience were tested by the task dependent ANOVA or Kruskal-Wallis test with all participants from temporal-position one and participants from categories reinforcement, non-reinforcement, and mixed-reinforcement. Participants of the category no-approach were excluded from the analysis of the CS+-Experience as they never approached the CS+ and therefore did not receive additional (non-)reinforcement.

##### Fear Conditioning and Recall

Subjective ratings were analyzed with a rmANOVA with stimulus and time as within factors.

The RR-change is the trial-wise readout based on the interpolated RR-timeseries. For that, we defined the baseline as the 5 s interval before stimulus onset. The RR-change was calculated as difference between the RR-value at 6 s after stimulus onset and the mean of the baseline. This readout was analyzed with a rmANOVA with stimulus and time as within factors. Due to missing data after technical problems with the device, we excluded five participants from the heart rate analyses within the Fear Conditioning and nine from the Recall task.

##### Touch the Enemy

The readout was calculated as the difference between the time to touch the first CS+ and the time to touch the first CS−. The time to touch was defined as the time from the end of the countdown until touching the CS by hand. Effects of temporal-position or CS+-Experience were tested with an ANOVA, one-sided one sample *t*-tests were used to test single groups for avoidance and independent *t*-tests were used for *post hoc* comparisons of the temporal-position one to the CS+-Experience categories. In this analysis we excluded four participants: one was starting before the countdown, one was running, one was an extreme outlier (time to touch > 15 s), and one lost the equipment during the task.

##### Path-Choice

For Path-Choice we counted the number of CS- passes before the first CS+ approach. A CS+ approach was defined as the participant passing the CS+, regardless of whether the participant continued walking or turned around and took the CS- path (which happened a few times only). With two directions (there and back) per trial and five trials in total, values between 0 and 10 are possible, where 0 means a CS+ approach at the very beginning and 10 means no CS+ approach at all. This readout is independent of whether the CS+ approach was reinforced or not. Since this results in a geometric distribution, the Kruskal-Wallis test was used to test for effects of temporal-position or CS+-Experience. We used the one-sided binomial test on the very first pass (readout > 0 or not) to test single groups for avoidance and the Mann-Whitney *U*-test for *post hoc* comparisons of the temporal-position one to the CS+-Experience categories.

##### Fishing

The readout of Fishing was defined as whether the participant started on the CS- or CS+ side and how long the participant stayed on that side. In order to make that robust against back and forth jumping on a single centered threshold, we defined a small, neutral band of approximately 1 m width in the middle between CS− and CS+ and analyzed at which side of the band the participant left. For this we calculated the difference between the distance from the participant to the CS+ and to the CS−. A difference of zero means equal distances to both stimuli and the participant was located on the bisecting line between them. If it exceeded the threshold of 1 m first, we defined it as avoidance behavior and measured the time until it was below −1 m. If it fell below −1 m first, we called it approach behavior and measured the time till it was above 1 m. To be able to distinguish between these two cases, we defined the approach-avoid time to be positive in the avoidance case and negative in the approach case. This definition leads to a symmetrically, but not normally distributed readout and the Kruskal-Wallis test was used to test for effects of temporal-position or CS+-Experience. To test single groups for avoidance, we also used the one-sided binomial test (readout > 0 or not) and the Mann-Whitney-U-test was used for *post hoc* comparisons of the temporal-position one to the CS+-Experience categories. In this analysis we excluded one participant due to misunderstanding the instructions, as reported in the interview after the experiment.

##### Across Tasks Analyses

The Friedman test (Friedman, 1937) was used to test for an effect of participant over all tasks. Similarly, we defined the *Rank-Sum* as the within participant over tasks sum of the within task over participants ranks and used it as measure for overall avoidance. We calculated the Spearman correlation between the tasks, the Rank-Sum, and questionnaires. Only correlations with uncorrected *p* < 0.05 are reported. A subsequent correction for multiple testing was performed with the Bonferroni procedure (corrected threshold: 0.05/(33^∗^4) = 0.00038). Sex differences were tested with the Mann-Whitney *U*-test. In these analyses, we excluded five participants which had been excluded in the analyses of any of the behavioral tasks, resulting in 50 participants (*M* = 24.4, *SD* = 23.5, range = 18–34, female: 27).

### Results

**FIGURE 2:**
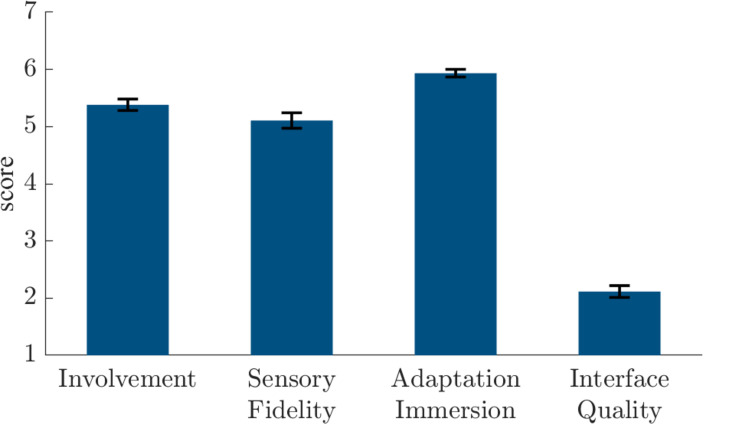
Presence Questionnaire 3 (Witmer et al., 2005) scores in the **quasi-experiment**.

Mean electrical shock current was 3.1 mA (*SD* = 1.2 mA). Participants rated it after the experiment as unpleasant (*M* = 6.3, *SD* = 1.4, scale = 1–10). As shown in Figure 2, participants reported high presence in the Presence Questionnaire 3 (*M* ± *SD*): involvement, 5.4 ± 0.7; sensory fidelity, 5.1 ± 1.0; adaptation immersion, 5.9 ± 0.5; interface quality, 2.1 ± 0.8. Moreover, they reported only slight side effects in the Simulator Sickness Questionnaire: total score, 14.3 ± 14.8; nausea, 11.4 ± 12.5; oculomotor symptoms, 10.7 ± 13.1; disorientation, 16.7 ± 22.3.

#### Fear Conditioning and Recall

**FIGURE 3:**
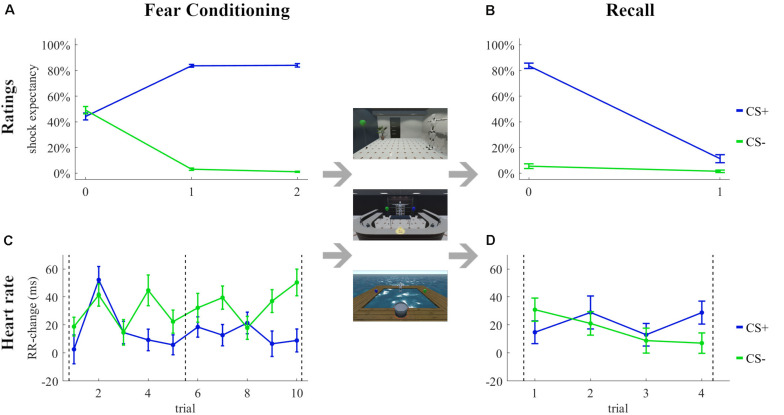
Subjective ratings **(A,B)** and changes in heart rate **(C,D)** during the Fear Conditioning **(A,C)** and Recall **(B,D)** tasks in the **quasi-experiment**. Rating 0 was the first subjective rating before the first trial. Dashed lines in **(C,D)** indicate the subjective ratings.

The shock expectancy ratings during the Fear Conditioning and Recall tasks are shown in Figures 3A,B, respectively. The analyses revealed a significant stimulus effect [*F*(1, 54) = 1083.8, *p* < 0.001, $\eta_{p}^{2}$ = 0.95, $\eta_{G}^{2}$ = 0.80], a time effect [*F*(2, 108) = 3.39, *p* = 0.04, $\eta_{p}^{2}$ = 0.06, $\eta_{G}^{2}$ = 0.02], and a stimulus × time interaction [*F*(2, 108) = 379.0, *p* < 0.001, $\eta_{p}^{2}$ = 0.88, $\eta_{G}^{2}$ = 0.71] during fear conditioning and a stimulus effect [*F*(1, 54) = 300.72, *p* < 0.001, $\eta_{p}^{2}$ = 0.85, $\eta_{G}^{2}$ = 0.66], a time effect [*F*(1, 54) = 436.55, *p* < 0.001, $\eta_{p}^{2}$ = 0.89, $\eta_{G}^{2}$ = 0.60], and a stimulus × time [*F*(1, 54) = 270.29, *p* < 0.001, $\eta_{p}^{2}$ = 0.83, $\eta_{G}^{2}$ = 0.54] interaction during recall.

The RR-changes during the Fear Conditioning and Recall tasks are shown in Figures 3C,D, respectively. The analyses revealed a stimulus effect [*F*(1, 49) = 13.63, *p* < 0.001, $\eta_{p}^{2}$ = 0.22, $\eta_{G}^{2}$ = 0.02], a trial effect [*F*(9, 441) = 3.12, *p* = 0.001, $\eta_{p}^{2}$ = 0.06, $\eta_{G}^{2}$ = 0.02], and a stimulus × trial interaction [*F*(9, 441) = 2.29, *p* = 0.02, $\eta_{p}^{2}$ = 0.04, $\eta_{G}^{2}$ = 0.02] during fear conditioning. None of the factors stimulus [*F*(1, 45) = 0.03, *p* = 0.86, $\eta_{p}^{2}$ = 0.00, $\eta_{G}^{2}$ = 0.00] and trial [*F*(3, 135) = 1.21, *p* = 0.31, $\eta_{p}^{2}$ = 0.03, $\eta_{G}^{2}$ = 0.01] or the interaction stimulus × trial [*F*(3, 135) = 1.61, *p* = 0.19, $\eta_{p}^{2}$ = 0.03, $\eta_{G}^{2}$ = 0.01] were significant during recall.

#### Touch the Enemy

**FIGURE 4:**
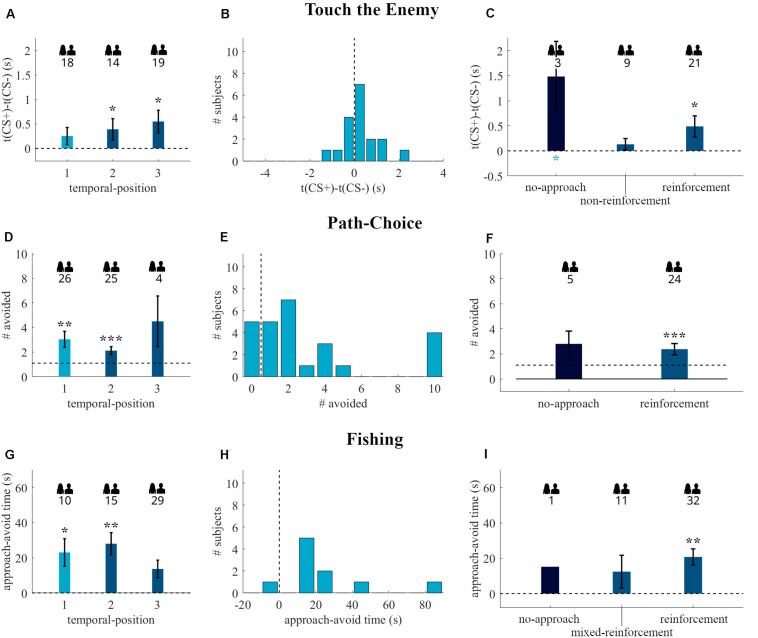
Behavioral readouts of the Touch the Enemy **(A–C)**, Path-Choice **(D–F)**, and Fishing **(G–I)** tasks in the **quasi-experiment**. The first column **(A,D,G)** depicts the readouts (mean and standard error) of the tasks grouped by the temporal-position in the individual task sequence. The second column **(B,E,H)** shows the histograms of participants that started with the respective task after fear conditioning (temporal-position one in **A,D,G**). The third column **(C,F,I)** contains the data (mean and standard error) of participants with temporal-position two and three, regrouped by the CS+-Experience categories. The dark blue bars are the no-approach categories, which were excluded in the CS+-Experience tests. Dashed lines indicate the border between approach (lower values) and avoidance (higher values) behavior. The numbers below the people indicate the number of participants included in the respective group. Black * above bars represent (uncorrected) significance levels of one-sample tests. Blue * below bars represent significance levels of comparison to temporal-position one. *p < 0.05; **p < 0.01; ***p < 0.001.

The time to touch difference increased with rising temporal-position (see Figure 4A), but our ANOVA revealed no significant effect of temporal-position [*F*(2, 48) = 0.55, *p* = 0.58, η^2^ = 0.02]. Single temporal-position analyses revealed no significant avoidance for temporal-position one [*t*(17) = 1.45, *p* = 0.08, *g*_1_ = 0.34], but an effect for temporal-position two [*t*(13) = 1.79, *p* = 0.05, *g*_1_ = 0.48] and three [*t*(18) = 2.40, *p* = 0.01, *g*_1_ = 0.55], see Figure 4B for the distribution of the readout for temporal-position one. The ANOVA revealed no effect of CS+-Experience [*F*(2, 45) = 0.77, *p* = 0.47, η^2^ = 0.03, see Figure 4C]. One sample *t*-tests showed no effect for non-reinforcement [*t*(8) = 1.14, *p* = 0.14, *g*_1_ = 0.38] and no-approach [*t*(2) = 2.11, *p* = 0.08, *g*_1_ = 1.22], by contrast, we observed a significant difference from zero for reinforcement [*t*(20) = 2.31, *p* = 0.02, *g*_1_ = 0.50].

#### Path-Choice

The Kruskal-Wallis test revealed no effect of temporal-position [X *^2^*(2, *N* = 55) = 1.31, *p* = 0.52]. As shown in Figure 4D, there was avoidance behavior in temporal-position one (*p* = 0.001, *N* = 26, 21 avoiders), and two (*p* < 0.001, *N* = 25, 21 avoiders), but not if Path-Choice was the last task (*p* = 0.31, *N* = 4, 3 avoiders). Figure 4E shows that 5 out of 26 participants with Path-Choice as first behavioral task directly approached the CS+, the others avoided the CS+ at least once. The grouping by the CS+-Experience shows no effect of additional reinforcement on avoidance behavior [*X ^2^*(1, *N* = 50) = 0.05, *p* = 0.82, see Figure 4F]. The binomial tests on single CS+-Experience categories showed an effect for reinforcement (*p* < 0.001, *N* = 24, 20 avoiders), but not for no-approach (*p* = 0.19, *N* = 5, 4 avoiders).

#### Fishing

**FIGURE 5:**
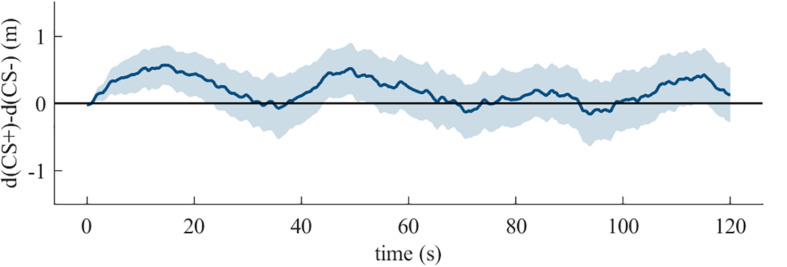
The mean with 95% confidence interval of the difference between the distances from the participant to the CS+ and the CS- by time for the duration of the Fishing task in the **quasi-experiment**.

Analyses of Fishing also revealed no effect of temporal-position [X *^2^*(2, *N* = 54) = 4.93, *p* = 0.09]. As shown in Figure 4G, there was avoidance behavior in temporal-position one (*p* = 0.01, *N* = 10, 9 avoiders), and two (*p* = 0.004, *N* = 15, 13 avoiders), but not if it was the last task (*p* = 0.07, *N* = 29, 19 avoiders). Nine out of ten participants with Fishing as first behavioral task avoided the CS+ (see Figure 4H). The grouping by the CS+-Experience (see Figure 4I) shows no effect of additional reinforcement on avoidance behavior [X *^2^*(2, *N* = 53) = 1.88, *p* = 0.39]. The binomial tests on single CS+-Experience categories showed an effect for reinforcement (*p* = 0.001, *N* = 32, 25 avoiders), but not for mixed-reinforcement (*p* = 0.50, *N* = 11, 6 avoiders) and no-approach (*p* = 0.50, *N* = 1, 1 avoiders). The temporally dynamic analysis showed that avoidance behavior was confined to the first twenty seconds (see Figure 5).

#### Across Tasks Analyses

The Friedman test revealed an effect of participant [${\text{X}}_{F}^{2}$(49) = 74.90, *p* = 0.01]. The Spearman correlations between the questionnaires and the readouts of the behavioral tasks are shown in Table 1. We found one significant correlation between cognitive concerns (ASI) and Path-Choice (*r*_*s*_ = 0.30, *p* < 0.05), but this did not survive correction for multiple testing. No (uncorrected) significant correlations were found with any of the other scales from the pre- or post-experiment questionnaires. The results of the sex comparisons are listed in Table 2 and shown in Supplementary Figure 1.

**TABLE 1:** Spearman correlations between questionnaires and behavioral tasks in the **quasi-experiment**.

		Value		Correlation							
		*M*	SD	Touch the Enemy		Path-Choice		Fishing		Rank-Sum	
						
				*r*_*s*_	*p*	*r*_*s*_	*p*	*r*_*s*_	*p*	*r*_*s*_	*p*
Task	Touch the enemy	0.35	0.79								
	Path-Choice	2.54	2.45	0.16	0.27						
	Fishing	19.12	26.71	0.23	0.11	0.41	0.003				
	Rank-Sum	76.50	31.00	**0.64**	<0.001	**0.73**	<0.001	**0.73**	<0.001		
	Age	24.42	4.20	–0.20	0.17	0.22	0.12	**0.30**	0.04	0.11	0.44
Valence rating	Shock (US)	6.22	1.39	–0.03	0.86	**0.36**	0.01	0.21	0.14	0.27	0.06
	CS+	–3.10	1.75	–**0.31**	0.03	–**0.35**	0.01	–**0.38**	0.007	–**0.48**	<0.001
	CS-	3.96	1.65	0.25	0.08	0.09	0.51	0.20	0.17	0.22	0.12
	CS- minus CS+	7.06	3.01	**0.36**	0.01	0.26	0.07	**0.38**	0.007	**0.44**	0.001

*Bold = p < 0.05.*

**TABLE 2:** Sex comparisons of the behavioral tasks with test statistic and *p*-values of the Mann-Whitney *U*-test and Cohens U3 effect sizes.

	Male (*N* = 22)		Female (*N* = 28)		Test		
	*M*	*SD*	*M*	*SD*	U	P	U3
Touch the enemy	0.3	0.8	0.4	0.8	292.0	0.764	0.55
Path-Choice	2.4	2.2	2.6	2.7	310.5	0.964	0.50
Fishing	15.3	26.9	22.1	26.7	251.0	0.272	0.73
Rank-Sum	73.3	29.1	79.0	32.7	275.5	0.532	0.64

### Discussion

In this quasi-experiment, we tested whether avoidance behavior induced by differential fear conditioning can be quantified by three behavioral tasks. Moreover, we tested the influence of additional reinforcement on this avoidance behavior.

Participants subjectively learned the contingency between the CSs and the US and this was still manifest in the Recall task after the behavioral tasks. Also, there was a significant increase in heart rate during the CS+ compared to the CS-. We observed no avoidance behavior in the behavioral approach task (Touch the Enemy), if it immediately followed the Fear Conditioning task. After additional reinforcement, participants showed avoidance behavior in this task. With the behavioral forced-choice (Path-Choice) and search tasks (Fishing), we observed avoidance behavior independent of additional reinforcement. In addition, we found that the occurrence of a non-reinforced trial in a preceding task eliminated avoidance behavior in the behavioral search task.

This quasi-experiment had multiple manipulations between the runs and no randomization between groups. The idea of analyzing the effect of additional reinforcement with categories of CS+-Experience emerged during the quasi-experiment. Due to this design, one could argue that the effects are to some extent confounded by recruiting time and other factors.

To examine these possibilities, we ran a confirmatory experiment, in which we randomly assigned participants to different task-orders, with counter-balanced reinforcement/non-reinforcement to ensure that there were enough participants in the relevant CS+-Experience categories. Additionally, we used a more intense US, consisting of a 2 s female voice scream together with three consecutive electrical shocks, since avoidance effects were rather small for some tasks and correlational analyses suggested a weak correlation between CS valence and avoidance behavior.

## Experiment

### Materials and Methods

The procedure of the experiment was largely identical to the quasi-experiment with the following modifications:

We measured 77 participants in this experiment: 3 of them dropped out due to technical issues with Unity, 1 canceled at the very beginning due to dizziness in iVR, and 1 was removed from analysis, as he reported to hardly perceive the electrical shocks. The excluded participants were replaced with new participants until the predefined 72 participants (*M* = 24.2, *SD* = 4.4, range = 18–34, female: 40) were reached. Participants were randomly assigned to 12 equal-sized groups with 6 different task orders and whether the CS+ approach in the first task was reinforced or not. In the second and third behavioral task a CS+ approach was always reinforced. One participant walked through the table in the Path-Choice task and was excluded from analyses of that task, as well as the across tasks analyses. In the heart rate analyses, we excluded 8 participants in the Fear Conditioning task and 11 participants in the Recall task, due to missing data after technical problems with the device.

We increased the intensity of the US as the effects in the quasi-experiment were rather small and we observed a weak correlation between avoidance behavior and CS valence. The US was a 95 dB female voice scream (first 2 s of no. 276 in IADS-2, Bradley and Lang, 2007), played simultaneously with three consecutive 100 ms electrical shocks (400 and 700 ms breaks). The calibration of a single electrical shock was identical to the quasi-experiment. Participants received the combined US the first time during the Fear Conditioning task.

The Tutorial was extended by a short scream habituation after the calibration of the electrical shock: Participants were informed that beside the electrical shock they will also hear a female voice scream. They heard it once alone and rated its valence afterward on the same scale as the electrical shock. In the Fear Conditioning task, we adapted the rating question for the new US to “How likely does an unpleasant stimulus occur for that object?” In the Path-Choice task, we moved the balloons next to the table in the center and introduced a pause of 2 s between finishing the trial and starting the next one. This was done to increase salience and recognition of the balloons to avoid incidental approaches of the CS+ after swapping of the CSs. In the Fishing task, the handle of the hand-net was shortened from 0.75 to 0.35 m to encourage more movement.

At the end, after the virtual reality experience, we added the iGroup Presence Questionnaire (Schubert et al., 1999) to the surveys to improve comparability to other studies.

### Results

**FIGURE 6:**
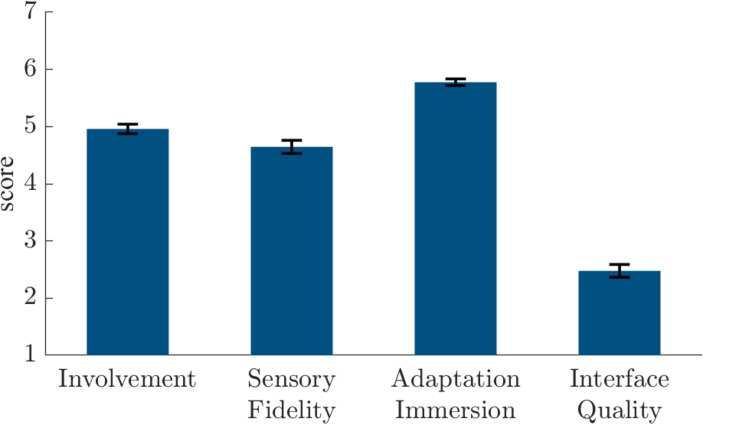
Presence Questionnaire 3 (Witmer et al., 2005) scores in the **experiment**.

Mean electrical shock current was 3.0 mA (*SD* = 1.1 mA). Participants rated it after the experiment as unpleasant (*M* = 7.2, *SD* = 1.3, scale = 1–10). The scream alone was perceived as less unpleasant (*M* = 3.7, *SD* = 2.1, scale = 1–10), while the combination of scream and electrical shocks were rated with a mean of 6.7 (*SD* = 1.5, scale = 1–10). We further analyzed potential differences in habituation according to US intensity (see Supplementary Material). As shown in Figure 6, participants reported high presence in the Presence Questionnaire 3 (*M* ± *SD*): involvement, 5.0 ± 0.7; sensory fidelity, 4.6 ± 1.0; adaptation immersion, 5.8 ± 0.5; interface quality, 2.5 ± 0.9. In the iGroup Presence Questionnaire they also reported high presence: general presence, 4.2 ± 1.3; spatial presence, 4.5 ± 0.9; involvement, 3.9 ± 1.2; experienced realism, 2.5 ± 1.1. Moreover, they reported only slight side effects in the Simulator Sickness Questionnaire: total score, 19.8 ± 21.0; nausea, 19.9 ± 20.7; oculomotor symptoms, 12.4 ± 13.9; disorientation, 21.8 ± 31.2.

#### Fear Conditioning and Recall

**FIGURE 7:**
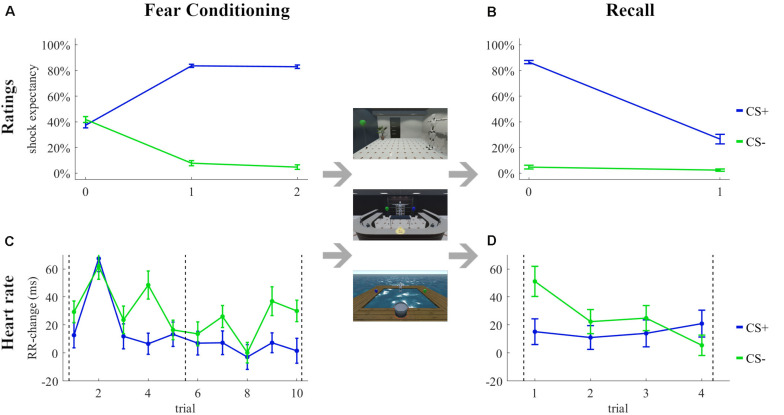
Subjective ratings **(A,B)** and changes in heart rate **(C,D)** during the Fear Conditioning **(A,C)** and Recall **(B,D)** tasks in the **experiment**. Rating 0 was the first subjective rating before the first trial. Dashed lines in **(C,D)** indicate the subjective ratings.

The analyses of the shock expectancy ratings during the Fear Conditioning (Figure 7A) and Recall (Figure 7B) tasks revealed a significant stimulus effect [*F*(1, 71) = 897.2, *p* < 0.001, $\eta_{p}^{2}$ = 0.93, $\eta_{G}^{2}$ = 0.73], a time effect [*F*(2, 142) = 5.75, *p* = 0.004, $\eta_{p}^{2}$ = 0.07, $\eta_{G}^{2}$ = 0.03], and a stimulus × time interaction [*F*(2, 142) = 421.4, *p* < 0.001, $\eta_{p}^{2}$ = 0.86, $\eta_{G}^{2}$ = 0.61] during fear conditioning and a stimulus effect [*F*(1, 71) = 591.0, *p* < 0.001, $\eta_{p}^{2}$ = 0.89, $\eta_{G}^{2}$ = 0.68], a time effect [*F*(1, 71) = 241.0, *p* < 0.001, $\eta_{p}^{2}$ = 0.77, $\eta_{G}^{2}$ = 0.43], and a stimulus × time interaction [*F*(1, 71) = 199.1, *p* < 0.001, $\eta_{p}^{2}$ = 0.74, $\eta_{G}^{2}$ = 0.39] during recall.

The RR-changes during the Fear Conditioning and Recall tasks are shown in Figures 7C,D, respectively. The analyses revealed a stimulus effect [*F*(1, 63) = 9.60, *p* = 0.003, $\eta_{p}^{2}$ = 0.13, $\eta_{G}^{2}$ = 0.01] and trial effect [*F*(9, 567) = 9.58, *p* < 0.001, $\eta_{p}^{2}$ = 0.13, $\eta_{G}^{2}$ = 0.05], but no stimulus × trial interaction [*F*(9, 567) = 1.56, *p* = 0.124, $\eta_{p}^{2}$ = 0.02, $\eta_{G}^{2}$ = 0.01] during fear conditioning. None of the factors stimulus [*F*(1, 60) = 1.76, *p* = 0.19, $\eta_{p}^{2}$ = 0.03, $\eta_{G}^{2}$ = 0.00] and trial [*F*(3, 180) = 1.97, *p* = 0.12, $\eta_{p}^{2}$ = 0.03, $\eta_{G}^{2}$ = 0.01] or the interaction stimulus × trial [*F*(3, 180) = 2.40, *p* = 0.07, $\eta_{p}^{2}$ = 0.04, $\eta_{G}^{2}$ = 0.02] were significant during recall.

#### Touch the Enemy

**FIGURE 8:**
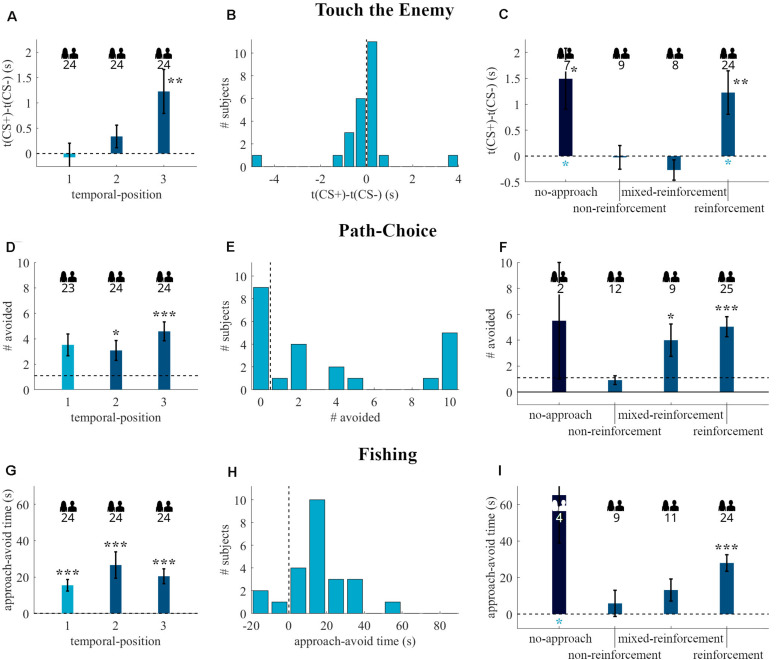
Behavioral readouts of the Touch the Enemy **(A–C)**, Path-Choice **(D–F)**, and Fishing **(G–I)** tasks in the **experiment**. The first column **(A,D,G)** depicts the readouts (mean and standard error) of the tasks grouped by the temporal-position in the individual task sequence. The second column **(B,E,H)** shows the histograms of participants that started with the respective task after fear conditioning (temporal-position one in **A,D,G**). The third column **(C,F,I)** contains the data (mean and standard error) of participants with temporal-position two and three, regrouped by the CS+-Experience categories. The dark blue bars are the no-approach categories, which were excluded in the CS+-Experience tests. The standard error of group no-approach in **(I)** is 26.3 s, and the error-bar extends to 92.6 s. Dashed lines indicate the border between approach (lower values) and avoidance (higher values) behavior. The numbers below the people indicate the number of participants included in the respective group. Black * above bars represent (uncorrected) significance levels of one-sample tests. Blue * below bars represent significance levels of comparison to temporal-position one. *p < 0.05; **p < 0.01; ***p < 0.001.

The ANOVA revealed an effect of temporal-position [*F*(2, 69) = 4.20, *p* = 0.019, η^2^ = 0.11] and, as depicted in Figure 8A, the time to touch difference increased with rising temporal-position. The t-tests on the single temporal-positions revealed no significant avoidance for temporal-position one [*t*(23) = −0.27, *p* = 0.61, *g*_1_ = −0.06, see Figure 8B] and two [*t*(23) = 1.51, *p* = 0.07, *g*_1_ = 0.31], but if this was the last task, avoidance behavior could be observed [*t*(23) = 2.82, *p* = 0.005, *g*_1_ = 0.58]. The ANOVA revealed an effect of CS+-Experience [*F*(3, 61) = 3.76, *p* = 0.02, η^2^ = 0.16, see Figure 8C]. The t-tests on the single CS+-Experience categories revealed no time to touch difference significantly higher than zero for non-reinforcement [*t*(8) = −0.11, *p* = 0.54, *g*_1_ = −0.04] and mixed-reinforcement [*t*(7) = −1.38, *p* = 0.89, *g*_1_ = −0.49] but we observed significant avoidance behavior in the categories reinforcement [*t*(23) = 2.92, *p* = 0.004, *g*_1_ = 0.60] and no-approach [*t*(6) = 2.53, *p* = 0.02, *g*_1_ = 0.96]. The independent *t*-tests between temporal-position one and the CS+-Experience categories revealed no increase for non-reinforcement [*t*(31) = −0.10, *p* = 0.92, Δ = −0.04] and mixed-reinforcement [*t*(30) = 0.39, *p* = 0.70, Δ = 0.14], however, the comparisons revealed an significant increase in the time to touch difference for no-approach [*t*(29) = −2.60, *p* = 0.01, Δ = −1.12], and reinforcement [*t*(46) = −2.58, *p* = 0.01, Δ = −0.94].

#### Path-Choice

Analyses of Path-Choice revealed no effect of temporal-position [*X ^2^*(2, *N* = 71) = 4.49, *p* = 0.11]. As shown in Figure 8D, there was no avoidance behavior in temporal-position one (*p* = 0.20, *N* = 23, 14 avoiders), but there was an effect, when the task was in second (*p* = 0.01, *N* = 24, 18 avoiders) or third place (*p* < 0.001, *N* = 24, 22 avoiders). Fourteen out of 23 participants with Path-Choice as first behavioral task avoided the CS+ at least once (see Figure 8E). The analysis by CS+-Experience (see Figure 8F) revealed an effect of categories [*X ^2^*(3, *N* = 69) = 13.42, *p* = 0.004]. The binomial tests on single categories revealed an effect for reinforcement (*p* < 0.001, *N* = 25, 23 avoiders) and mixed-reinforcement (*p* = 0.02, *N* = 9, 8 avoiders), but we did not observe significant avoidance behavior for non-reinforcement (*p* = 0.39, *N* = 12, 7 avoiders) and no-approach (*p* = 0.25, *N* = 2, 2 avoiders). The comparison of the temporal-position one to the CS+-Experience categories revealed no significant difference for no-approach [*U*(n_1_ = 23, n_2_ = 2) = 16, *p* = 0.54, *U3* = 0.74], non-reinforcement [*U*(n_1_ = 23, n_2_ = 12) = 149, *p* = 0.14, *U3* = 0.41], mixed-reinforcement [*U*(n_1_ = 23, n_2_ = 9) = 84.5, *p* = 0.43, *U3* = 0.50], and reinforcement [*U*(n_1_ = 23, n_2_ = 25) = 204, *p* = 0.08, *U3* = 0.65].

#### Fishing

**FIGURE 9:**
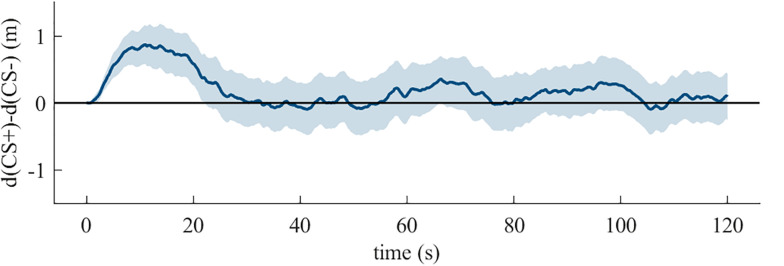
The mean with 95% confidence interval of the difference between the distances from the participant to the CS+ and the CS- by time for the duration of the Fishing task in the **experiment**.

The Kruskal-Wallis test revealed no effect of temporal-position [*X ^2^*(2, *N* = 72) = 1.85, *p* = 0.40]. As shown in Figure 8G, there was avoidance behavior regardless of whether this task was in first (*p* < 0.001, *N* = 24, 21 avoiders), second (*p* < 0.001, *N* = 24, 21 avoiders), or third (*p* < 0.001, *N* = 24, 20 avoiders) place. Figure 8H shows that 21 out of 24 participants with Fishing as first behavioral task avoided the CS+, whereas the other participants approached the CS+ at the beginning. The analysis on the CS+-Experience showed no group effect [*X ^2^*(3, *N* = 68) = 5.97, *p* = 0.11, see Figure 8I]. The binomial tests revealed significant avoidance behavior for the category reinforcement (*p* < 0.001, *N* = 24, 23 avoiders), but not for no-approach (*p* = 0.06, *N* = 4, 4 avoiders), non-reinforcement (*p* = 0.25, *N* = 9, 6 avoiders), and mixed-reinforcement (*p* = 0.11, *N* = 11, 8 avoiders). The temporally dynamic analysis again showed that avoidance behavior was confined to the first twenty seconds (see Figure 9).

#### Across Tasks Analyses

The Friedman test revealed an effect of participant [X ^2^(70) = 101.07, *p* < 0.01]. The Spearman correlations between the questionnaires and the readouts of the behavioral tasks are shown in Table 3. We found nominally significant correlations between nausea (SSQ) and Path-Choice (r_*s*_ = 0.26, *p* < 0.05), between sensory fidelity (PQ3) and Path-Choice (r_*s*_ = 0.30, *p* < 0.05), between interface quality (PQ3) and Fishing (r_*s*_ = 0.26, *p* < 0.05), and between somatic concerns (ASI) and Fishing (r_*s*_ = 0.26, p < 0.05), but none survived correction for multiple testing. No (uncorrected) significant correlations were found with any of the other scales from the pre- or post-experiment questionnaires. The results of the sex comparisons are listed in Table 4 and shown in Supplementary Figure 4.

**TABLE 3:** Spearman correlations between questionnaires and behavioral tasks in the **experiment**.

		Value		Correlation							
		*M*	SD	Touch the enemy		Path-Choice			Fishing	Rank-Sum	
				*r*_*s*_	*p*	*r*_*s*_	*p*	*r*_*s*_	*p*	*r*_*s*_	*p*
Task	Touch the enemy	0.51	1.67								
	Path-Choice	3.73	3.86	0.17	0.15						
	Fishing	21.04	25.40	**0.24**	0.04	**0.25**	0.04				
	Rank-Sum	108.00	42.61	**0.67**	<0.001	**0.67**	<0.001	**0.72**	<0.001		
	Age	24.25	4.37	0.15	0.21	**0.31**	0.008	0.04	0.76	0.22	0.07
Valence rating	Shock	7.21	1.32	0.09	0.48	0.21	0.07	0.23	0.05	**0.27**	0.02
	Scream	3.68	2.06	–0.03	0.80	0.19	0.12	0.15	0.22	0.13	0.29
	Combined (US)	6.72	1.54	0.18	0.14	**0.25**	0.04	**0.34**	0.004	**0.38**	0.001
	CS+	–3.76	1.25	–0.04	0.72	–**0.27**	0.02	–0.08	0.50	–0.21	0.07
	CS-	3.62	1.69	0.02	0.85	–0.18	0.14	–0.11	0.38	–0.11	0.38
	CS- minus CS+	7.38	2.07	0.07	0.57	0.02	0.90	–0.03	0.82	0.06	0.63

*Bold = p < 0.05.*

**TABLE 4:** Sex comparisons of the behavioral tasks with test statistic and *p*-values of the Mann-Whitney-U test and Cohens U3 effect sizes.

	Male (N = 31)		Female (N = 40)		Test		
	*M*	*SD*	*M*	*SD*	*U*	*p*	U3
Touch the enemy	0.3	0.9	0.7	2.1	621.0	0.993	0.35
Path-Choice	2.2	2.9	4.9	4.1	382.5**	0.004	0.77
Fishing	20.1	26.2	21.8	25.0	613.5	0.943	0.48
Rank-Sum	100.2	41.6	114.1	42.9	498.0	0.159	0.68

**p < 0.01.

### Discussion

In the confirmatory experiment, we tested the effect of differential fear conditioning with a more intense US on avoidance behavior in the same three tasks. Moreover, we manipulated whether a CS+ approach was reinforced in the first task after the Fear Conditioning task and tested the effect of this additional reinforcement on avoidance behavior in subsequent tasks. The results of the subjective ratings and heart rate analyses again demonstrated that participants learned the CS-US contingency and showed a physiological fear response to the CS+. In our behavioral approach task (Touch the Enemy), we observed avoidance behavior only in the case of additional reinforcement (without any non-reinforcement) in a preceding task, but not if it followed right after the Fear Conditioning task. Similarly, in the behavioral forced-choice task (Path-Choice), we also observed avoidance behavior only in case of an additional reinforcement in a preceding task, but not if it followed right after the Fear Conditioning task. In the behavioral search task (Fishing), we observed avoidance behavior in both cases, independent of further reinforcement. However, if there was a CS+ approach without reinforcement in any preceding task, avoidance behavior was also no longer present in this task.

## General Discussion

In the quasi-experiment we performed four runs, analyzed them together to explore initial effects, and tested the robustness of these effects in a subsequent confirmatory experiment. The results of the second experiment provided a replication for the effects of our behavioral approach task and behavioral search task, however for the behavioral forced-choice task, the effects did not replicate to the same extent. In the confirmatory experiment, we used a more intense US to increase avoidance behavior, as analyses in the quasi-experiment showed correlations between stimulus valence and avoidance behavior. However, stimulus valence and avoidance behavior were only marginally increased in the confirmatory experiment, indicating a weaker influence of US intensity than assumed and other factors might be more important, which will be discussed below.

We compared behavioral tasks with varying instructions, degrees of freedom, and high or low task related relevance of the stimuli on the sensitivity of the tasks to detect experimentally induced avoidance behavior. The behavioral search task had the highest degrees of freedom, a gaming element (“catching a fish”) and no task relevance of the CSs. Almost all participants avoided the CS+ initially, even in the case of no additional reinforcement when it followed right after the Fear Conditioning task. While they were fishing, all participants moved to the CS- side at first and remained there, on average for 20 s. These findings show that this task is sensitive for avoidance behavior after fear conditioning in healthy human participants. Our behavioral approach task had the lowest degrees of freedom and the CSs were relevant for the task, as participants were instructed to touch them. This task did not result in avoidance behavior directly after the Fear Conditioning task. Instead, we only observed avoidance behavior after additional reinforcement in previous scenarios, pointing toward its necessity. The results from the behavioral forced-choice task suggest that this task falls in between the other two. Interestingly, as participants had to pass the CSs in this task, the task relevance of the CSs was higher than in the search task but lower than in the approach task. Furthermore, in the quasi-experiment the stimuli were placed outside the paths, but in the experiment, they were placed in such a manner that participants had to walk around them. In this way the task relevance of the CSs was increased, which might explain the absence of avoidance behavior after fear conditioning in the experiment. We speculate that the lower the task relevance of the CSs, the more likely one observes avoidance behavior in that task. Other potential factors influencing the sensitivity of a task to detect avoidance behavior could be participants’ degree of freedom, gamification or the cover story. However, it is open whether these factors have a direct effect on sensitivity or whether the effects are mediated by the task relevance of the stimuli.

This leads us to speculate that the more relevant the CSs are to the task, the more “cognitive” the avoidance behavior. In an unstructured *post hoc* interview, in which we asked participants why they behaved as they did, some participants reported that uncertainty in shock expectancy led to mistrust of the experimental procedure (“Now they probably changed the contingencies.”) and testing behavior, resulting in an approach of the CS+. Such cognitions can be instilled by interpretations of the instructions and can lead to testing behavior of individually developed hypotheses. We hypothesize that differences in cognitions explain a high proportion of the variance in individual differences in behavior. However, it is difficult to objectively and reliably measure cognitions directly, without affecting them; nevertheless, the individual creation and testing of hypotheses should be considered in future research, possibly initially with more qualitative approaches.

Avoidance behavior is often weighed against other behavioral alternatives with competing motivations. These comprise the situational evaluation of likelihoods, including information of the efficacy of responses and the cost of avoidance (Sheynin et al., 2015; Servatius, 2016). The presented behavioral tasks differ in the number of possible behaviors, the efficacy of participants’ responses in avoiding the US, as well as the cost of avoidance. Thus, various forms of avoidance behavior across the tasks can be expected. In line with that, the cross-correlations among the behavioral tasks showed only one weak correlation, between the behavioral search and forced-choice tasks, in both experiments. This relationship was also evident in the pattern of the CS+-Experience of the tasks: In all tasks, we observed avoidance behavior in the reinforcement category but not in the non-reinforcement category. However, in the behavioral search and forced-choice tasks, we also observed avoidance behavior in the mixed-reinforcement category, which was not the case for our behavioral approach task. These findings suggest that the observed avoidance behavior might be rather task-specific, and the different CS+-Experience patterns indicate that avoidance in different tasks might be based on different learning mechanisms. This is in line with the Principles of Avoidance Learning (Krypotos et al., 2015): According to this theory, Pavlovian conditioning is sufficient for action tendencies and the necessity of instrumental conditioning is depending on the type of the behavioral response.

Another important question is whether reinforcement during the behavioral tasks can be interpreted as instrumental conditioning. Existing theories of avoidance learning (Krypotos et al., 2015) differ in the role of instrumental conditioning and the assumed reinforcer, but most assume positive reinforcement of the avoidance behavior. In our study, approach and avoidance behavior differed across the tasks, so it could be argued that no specific behavior was reinforced or at least that it was too generic. The effects we observed could have been due to additional reinforcement functioning as generalization of CS-US contingencies across contexts and scenes. In our opinion, the most likely interpretation is that the reinforcement strengthened the CS-US contingencies. Even though we used a high reinforcement rate during fear conditioning and asked for the probability of receiving a shock, the ratings could have reflected only the past reinforcement rate instead of a future expectation with included uncertainty. We speculate that, in addition to the only slight increase in the reinforcement rate, the uncertainty was greatly reduced, and thus the CS-US contingency was strengthened. This impression was supported by many comments in the *post hoc* interview regarding the uncertainty (e.g., “It could have been that there was no shock”, “Was not sure if there would be a shock.”).

The manipulation of reinforcement during the behavioral tasks also enabled us to investigate the effect of non-reinforcement trials on behavior in subsequent tasks. These non-reinforcement trials can be interpreted as an extinction phase. In line with literature on extinction learning (Milad and Quirk, 2012) and exposure therapy (Foa and Kozak, 1986; Craske et al., 2014), we observed that non-reinforcement trials in one task extinguished avoidance behavior in the subsequent task, independent of the type of the tasks. However, this acquisition of avoidance might be an adaptive mechanism, whereas pathological anxiety could be better modeled by the persistence of avoidance behavior after the threat is gone. Our results indicate that the tasks can also be used to investigate this pathological avoidance. To do so, future work could add a new extinction task after the Fear Conditioning task or compare avoidance behavior after non-reinforcement in the behavioral search task to avoidance behavior after non-reinforcement in the approach task, examining the effects of explicit vs. implicit approach.

Beside the inter-task differences, we also observed robust within-task inter-individual differences. The participants were distributed on a wide range of avoidance intensities in each task, even in the behavioral search task, in which most participants avoided the CS+ in the beginning. Such dimensional behavioral expressions of avoidance might be useful in translating basic science to psychopathology (Servatius, 2016; Krypotos et al., 2018). Interestingly, the distribution appeared bimodal in the forced-choice task, indicating subtypes of behavior rather than a continuous dimension. Furthermore, we found some consistent individual differences across tasks, indicating that some participants show high avoidance behavior in all tasks and some participants show low avoidance behavior in all tasks, even though the correlations between the tasks were weak at best. This raises the question whether these differences are driven by trait factors. Pittig et al. (2020) listed trait anxiety, intolerance of uncertainty, anxiety sensitivity, neuroticism, and age as possible moderators of avoidance. However, our analyses did not show any robust correlations, which are present in both experiments, between avoidance behaviors and traits assessed by subjective questionnaires. Regarding sex differences, Sheynin et al. (2014) and Pittig et al. (2020) reported enhanced avoidance in female compared to male participants. In line with that, we found increased avoidance in female participants in the behavioral forced-choice task. However, we observed this difference only in that task, and even this effect was only present in the confirmatory experiment, but not in the quasi-experiment.

It remains therefore an open question, whether traits and sex explain much variance in individual differences in avoidance behavior, and how this phenomenon can be further explained. One possible direction for this question would be the comparison of healthy controls with patients. In our sample, we included only healthy participants, which limited the variance in the questionnaires. Extending the study protocol to patients with anxiety disorders would increase variance, and could also increase avoidance behavior, which might help explain individual differences.

The changing contexts might bring up the question whether they have led to a reduction of avoidance behavior, as participants might not automatically generalize from one scene to the next in iVR. We picked this approach as we were interested in cued fear conditioning. In animal research, this usually involves different contexts for different experimental phases to minimize contextual effects and maximize effects from CS-US pairing (Maren et al., 2013; Lonsdorf et al., 2017). In humans, this has been done only sometimes, due to methodological difficulties. It is plausible that procedures using the same context, as done in a monitor-based virtual environment study before (Greville et al., 2014), would have led to more robust avoidance behavior. In our study, however, it could have led to some difficulties in finding a one-size-fits-all solution for the various tasks and to transfer effects across scenes. For instance, during the search task, participants could have avoided the position where the CS+ was placed in the preceding task. One of the strengths of iVR is that context has become a factor that can more easily be experimentally manipulated, which opens the field for new research questions.

A limitation of the current study relates to CS+-Experience. According to its definition, it is task specific. This means, that the CS+-Experience for one behavioral task is based on participants’ behavior in the two other tasks. For instance, reinforcement before our behavioral approach task must occur in the behavioral search or forced-choice tasks, whereas reinforcement for the behavioral search task must occur in our behavioral approach or forced-choice tasks. Within our definition of CS+-Experience, the reinforcements in the different behavioral tasks are treated the same, but there could be a difference between the reinforcement when touching the CS+ as in our behavioral approach task, and the reinforcement when passing by the CS+ as in the behavioral search or forced-choice tasks. Nevertheless, if we compare two behavioral tasks, the context of their CS+-Experience has an overlap of 50%, as the third task is the same for both. Another limitation of what we defined as CS+-Experience is that it is based on participants’ behavior, which they could freely choose. A participant had to have approached the CS+ to be able to undergo non-reinforcement and/or reinforcement, and some did not. As less than 10% of participants were “full avoiders,” we can expect the effect of this confound on our results to be low. To overcome these difficulties, future research should consider inserting an instrumental conditioning task with no degrees of freedom after the Fear Conditioning task, but before the behavioral tasks. Another limitation relates to the motion of the CSs. During the Fear Conditioning task, the CSs were floating toward participants or into the air. However, in the behavioral search task, they swayed slightly in the wind and in our behavioral approach and forced-choice task, they did not move. It is possible that motion is a salient element of the stimuli, although the difference between a CS floating toward participants vs. minimal stationary swaying appears rather large.

In our study, we developed a new paradigm in iVR to experimentally induce and assess avoidance behavior. Participants were placed in the virtual scenes, could freely look around, saw a representation of their body, and navigated by naturalistic movements. This resulted in a very high presence with hardly any side effects, as well as naturalistic body movements and real-life behavior of participants within these artificial environments. The tasks of touching, choosing, and searching are common real-life behaviors. Therefore, the ecological validity of iVR appears high. Another advantage of this approach is that participants are confronted with the CS instead of the US, which might model a comparable process in anxiety disorders (Krypotos et al., 2015).

## Conclusion

To conclude, we observed avoidance behavior in all three tasks, probing different types of avoidance behavior. The behavioral approach and forced-choice tasks were sensitive to “strong” avoidance behavior after additional reinforcement, whereas the most sensitive task to detect avoidance behavior after fear conditioning was our behavioral search task, with low task relevance of the CSs, the highest degrees of freedom, and distraction by gamification elements.

## Data Availability Statement

All datasets presented in this study are included in the article/Supplementary Material.

## Ethics Statement

The studies involving human participants were reviewed and approved by the Faculty of Medicine at Ludwig Maximilian University of Munich. The patients/participants provided their written informed consent to participate in this study.

## Author Contributions

FB and VS designed the study. FB developed and implemented the tasks, recruited the participants, acquired and analyzed the data. FB and VS interpreted the data. FB wrote the manuscript under supervision of VS. All authors read and approved the submitted version.

## Conflict of Interest

The authors declare that the research was conducted in the absence of any commercial or financial relationships that could be construed as a potential conflict of interest.

## Supplementary Material

Supplementary Video 1 Video of the Touch the Enemy task.

Supplementary Video 2 Video of the Path-Choice task.

Supplementary Video 3 Video of the Fishing task.

Supplementary Data Sheet 1 Supplementary tables and figures.

Supplementary Data Sheet 2 Underlying data of the **quasi-experiment**.

Supplementary Data Sheet 3 Underlying data of the **experiment**.

Supplementary Data Sheet Unity package containing the virtual scenes.
