# Supplementary material for: Quantifying Human Avoidance Behavior in Immersive Virtual Reality
Source: Front Behav Neurosci. 2020 Sep 30;14:569899. doi: 10.3389/fnbeh.2020.569899 (PMC7554565; doi:10.3389/fnbeh.2020.569899)
Supplement: Supplementary Data Sheet 1 — Supplementary tables and figures. [file Data_Sheet_1.PDF]

Supplementary Material

1 Readout distributions

1.1 Quasi-Experiment

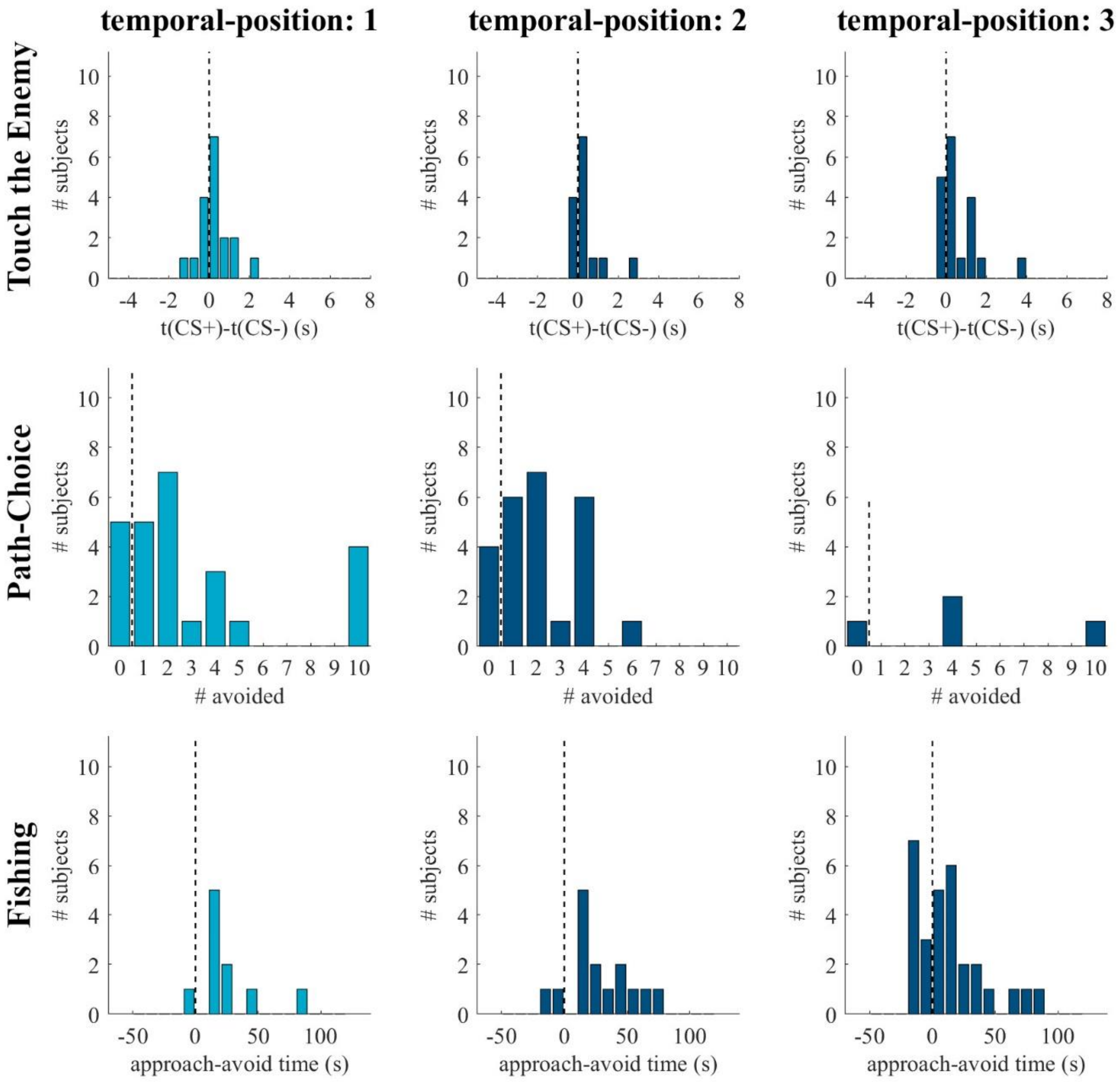

Supplementary Figure 1.

1.2 Experiment

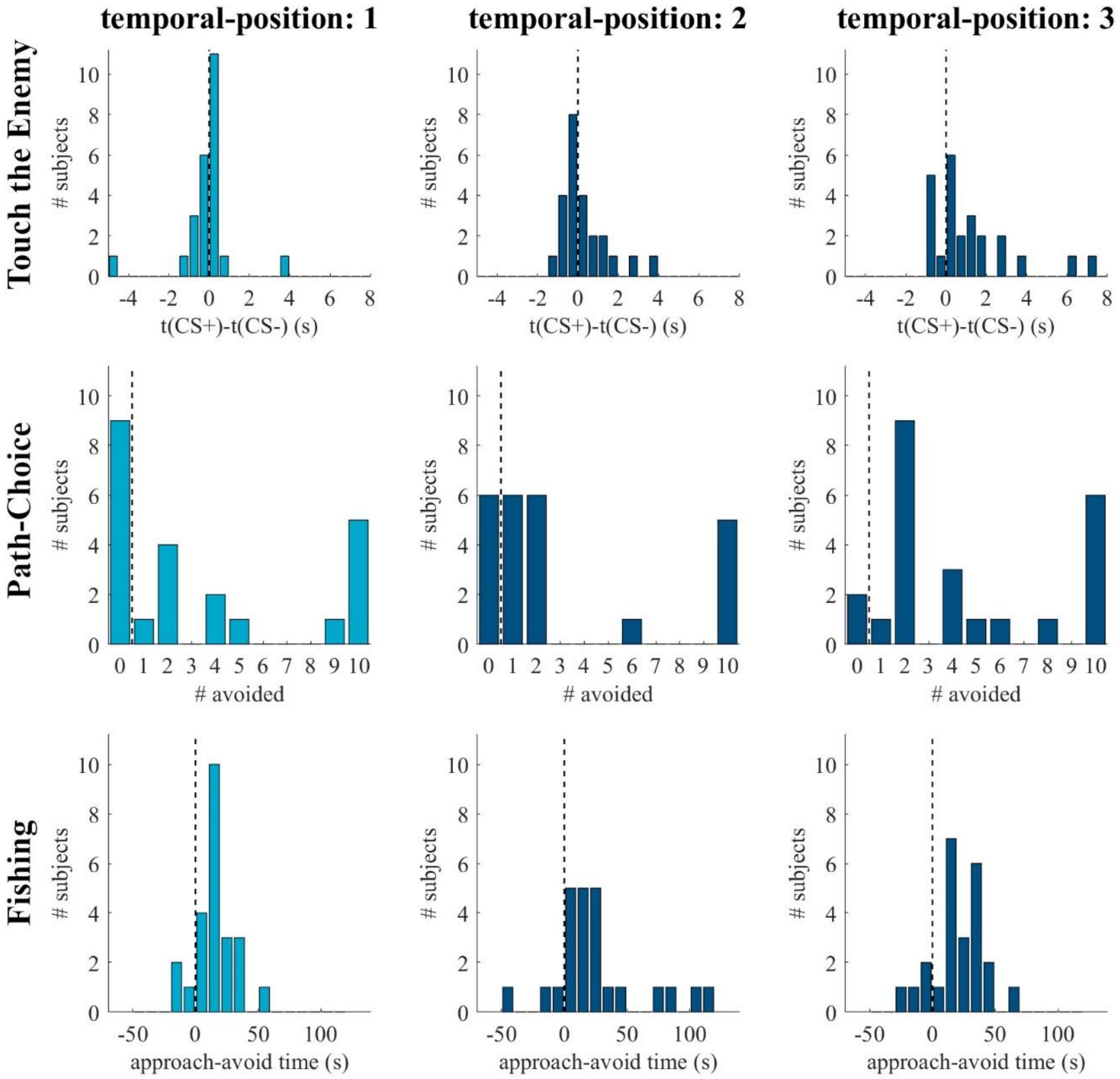

Supplementary Figure 2.

2 Sex Comparisons

2.1 Quasi-Experiment

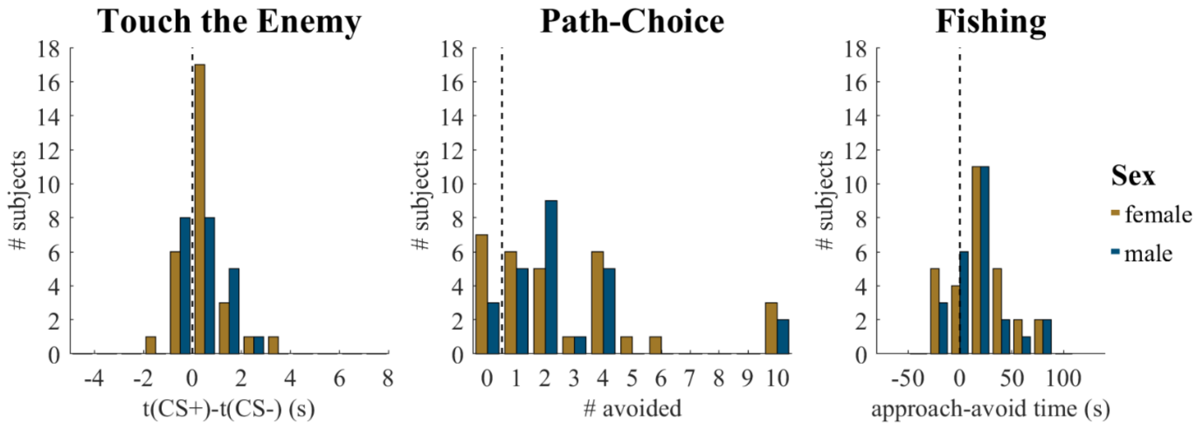

Supplementary Figure 3.

2.2 Experiment

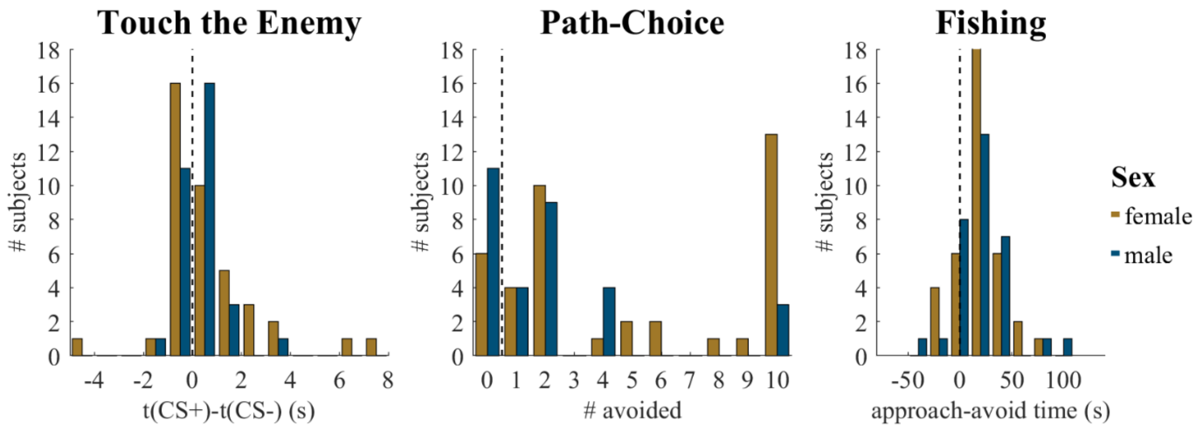

Supplementary Figure 4.

3 US habituation

US habituation is a common phenomenon in fear conditioning paradigms with electrical shocks. A US consisting of three shocks leads to more shocks in total and therefore might have led to stronger habituation, although the shocks followed each other with 400 ms and 700 ms breaks. Therefore, we analyzed our data to test this in the physiological, behavioral and subjective dimensions:

For the physiological dimension, we analyzed the heart rate during the fear conditioning task. Supplementary Figure 5 shows the baseline-corrected (mean of five seconds before CS onset) RR-interval by time for all eight reinforced CS+ trials of participants in the quasi-experiment with the lower intensity US (gray) and the experiment with the high intensity US (black). The red area indicates the occurrence of the US. For each reinforced trial, we calculated the mean of second 8 till 11 after stimulus onset as readout (blue area). In Supplementary Figure 6, these readouts are plotted as mean with standard error by the sequence of occurrence. It shows an increase (towards 0) and stabilization in the second block at around -20 ms. The outlier at the second US is probably due to arousal after the first (surprising) US. An rmANOVA with US-trial as within-factor and study as between-factor revealed an time effect of US-trial ($F(7,742) = 12.97$, $p < 0.001$), but no group effect of US-intensity ($F(1,106) = 0.09$) or interaction effect of US-trial * US-intensity ($F(7,742) = 0.59$). Similar results were obtained when US-trial 2 was excluded. We interpreted this as US-habituation during the fear conditioning task, however without differences between the quasi-experiment with lower US intensity and the experiment with higher US intensity.

For the behavioral dimension, we assumed a lower US valence will lead to more administered US events in the Fishing and Path-Choice tasks, as participants had the choice of approaching the CS+ or not. Combined with the expected stronger habituation, we would have expected to observe more US occurrences in these tasks the later they were in the temporal-position. To avoid the confounding effect of non-reinforced trials we focused on participants with CS+-Experience category reinforcement (n=39 in quasi-experiment and n=42 in the experiment). Supplementary Figure 7 shows the mean (+ standard error) number of applied US by task and temporal position. There was no indication for a temporal-position effect, which we interpret as there being no strong evidence in favor of habituation.

For the subjective dimension, we considered habituation as decreasing US valence by the number of perceived shocks and expected this to be present in the retrospective ratings of the electrical shock. Contradictorily, these ratings were slightly higher ($t(119) = 3.98$, $p < 0.001$) in the experiment (M = 7.2, SD = 1.3) than in the quasi-experiment (M = 6.3, SD = 1.4). This was in line with our impression from the qualitative interviews after the experiment where participants reported more of a sensitization than a habituation (“Over time the shocks became really annoying.”), although we cannot prove that direction either.

In summary, the results for the physiological, behavioral and subjective data show no difference in habituation between the single-shock US and the combined US. Therefore, a subsequent extinction is not likely. This conclusion is supported by persisting avoidance behavior during the tasks and in the recall data. After all, the subjective ratings and the heart rate data showed a clear differentiation between CS+ and CS- in the first recall trial (Figure 2 B,D and Figure 5 B,D in the manuscript).

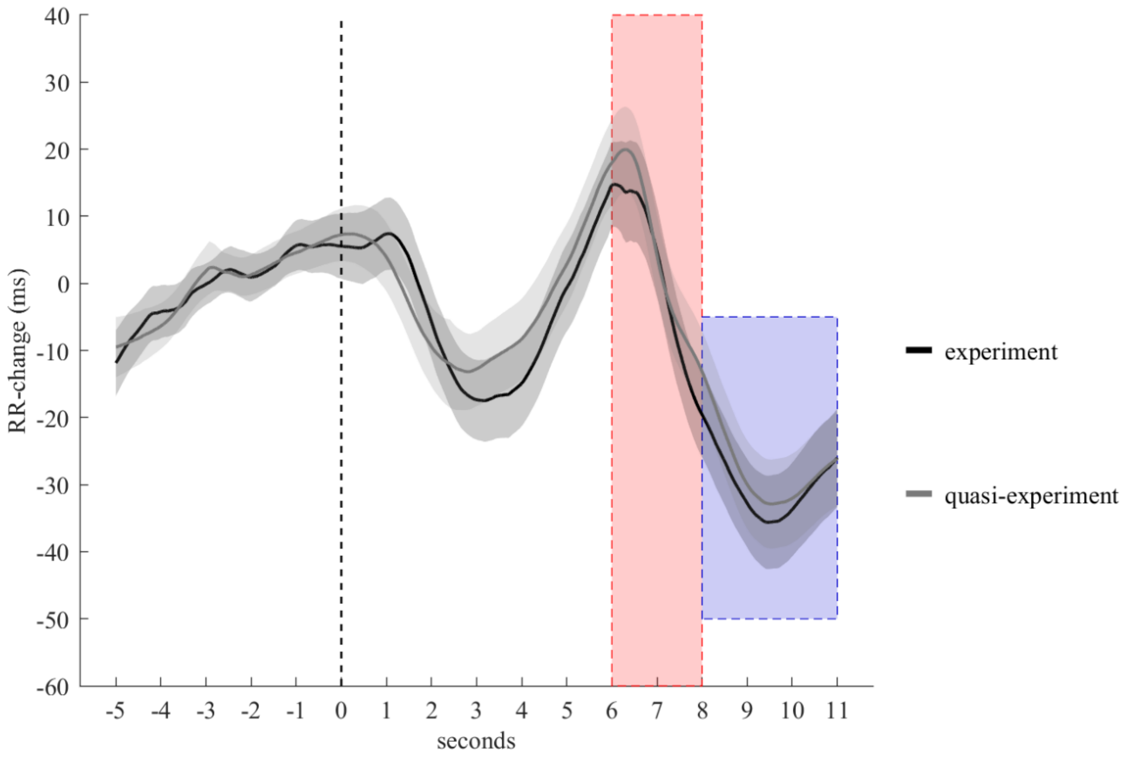

Supplementary Figure 5. Time course of the RR-change (mean and 95% CI) for the eight reinforced CS+ trials during fear conditioning. It was corrected by the mean during baseline (-5 s to 0 s). The black dashed line represents the stimulus onset at second 0. The US occurred at second 6 in the quasi-experiment and between seconds 6 and 8 in the experiment (red area). The mean during seconds 8 and 11 was used as readout for the US response in further analyses.

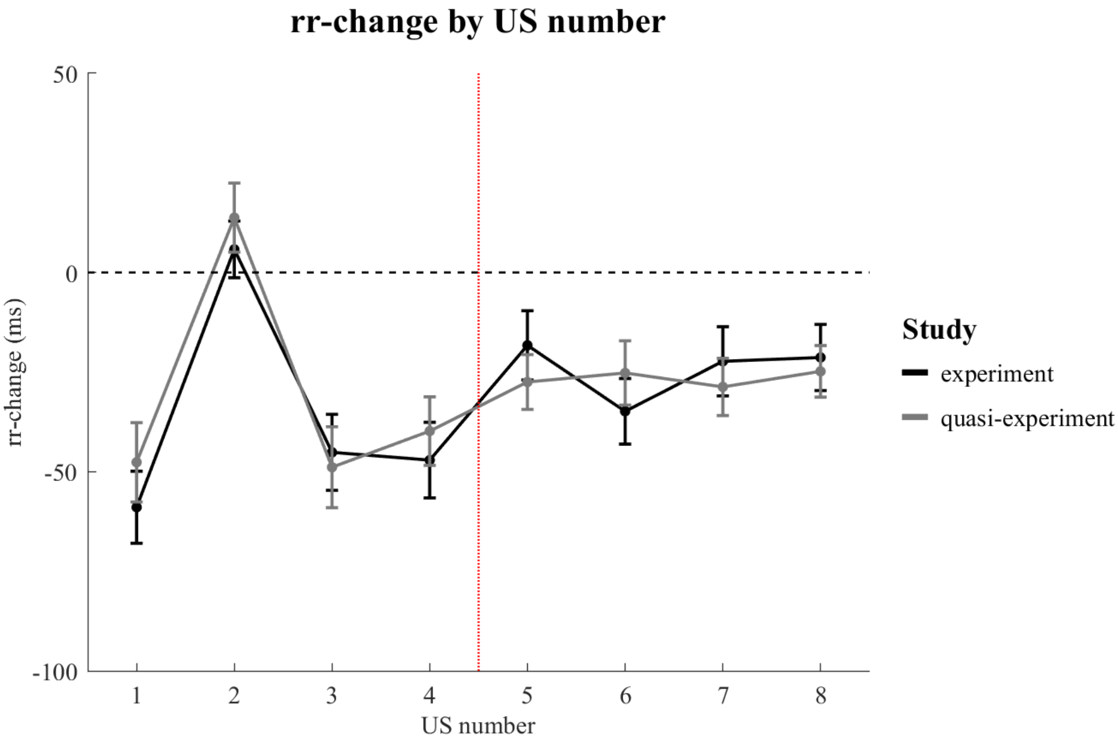

Supplementary Figure 6. Heart rate response (mean with standard-error) to the US by trial for all eight reinforced CS+ presentations during fear conditioning. Dotted red line represents the occurrence of the subjective rating.

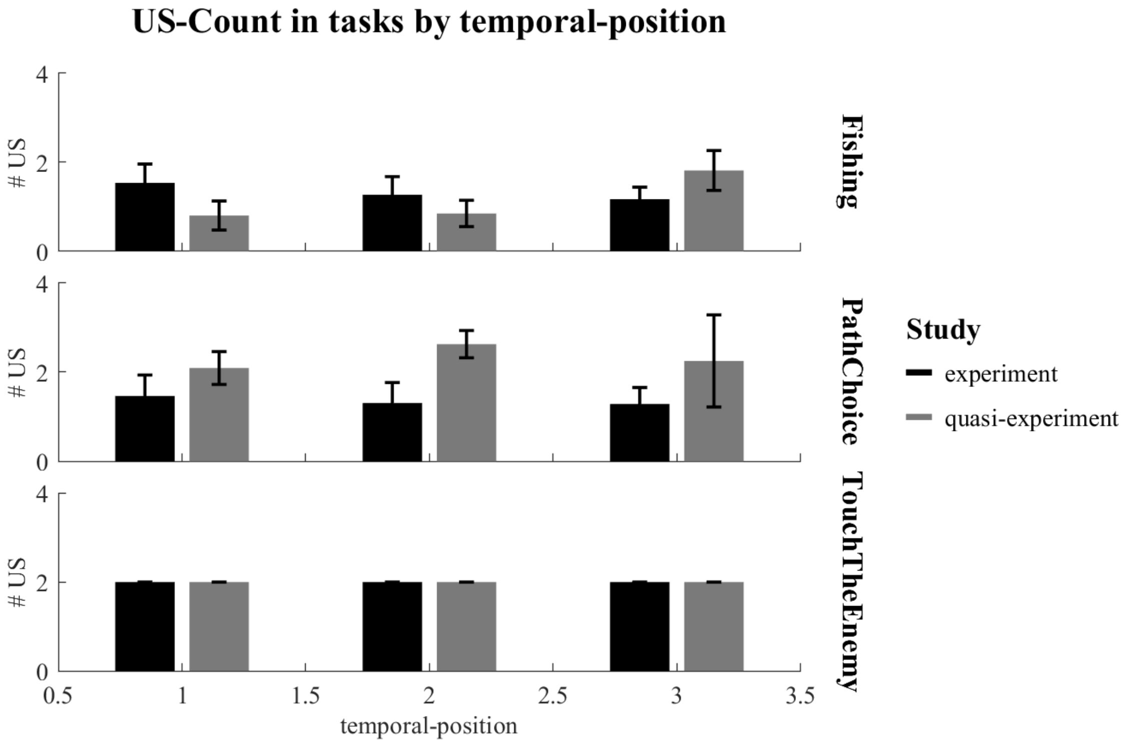

Supplementary Figure 7. Number of US-occurrences (mean with standard-error) during the behavioral tasks (rows) by the temporal-position of appearance in the individual task sequence.

4 Original Instructions during the Fear Conditioning task

Manuscript (translation)	Original instruction (German)
How likely does an electrical shock occur?<br><br>(quasi-experiment)	„Wie wahrscheinlich erfolgt ein Reiz?“
They received the instruction that in all following tasks the unpleasant stimuli may occur again.	„Bitte beachten Sie: In allen nun folgenden Aufgaben können weiterhin unangenehme Reize auftreten.“
How likely does an unpleasant stimulus occur for that object?<br><br>(experiment)	„Wie wahrscheinlich erfolgt bei diesem Objekt ein unangenehmer Reiz?“

5 Tables

Supplementary Table 1

Run	Participants		Fear-Conditioning	Behavioral Tasks							
	Number	Included	Balloon direction	Order	Touch the Enemy		Path-Choice			Fishing	
					Trials	reinforced	Balloon pos	Break after pos	reinforced	Hand-net length	Reinforced
**1**	14	12	towards	randomized	2x2	yes	outside	0 s	yes	long	yes
**2**	12	11	towards	fixed: PATH,TTE,FISH	3x2	66%	outside	0 s	no	long	yes
**3**	23	22	both	randomized	2x2	yes	outside	0 s	yes	long	yes
**4**	11	10	towards	fixed: TTE,PATH,FISH	2x2	yes	outside	0 s	yes	long	yes
**experiment**	77	72	towards	randomized	2x2	yes+no	table	2 s	yes+no	short	yes+no

5.1 Legend

Participants

Number	Number of participants who participated in the experiment.
Included	Number of participants included in the analyses.

Balloon direction

Towards	Balloons were floating towards the participant.
Up	Balloons were floating up in the air.
Both	Towards and up was counterbalanced between participants.

Trials

2x2	Two trials for each CS (alternating).
3x2	Three trials for each CS (alternating).

Reinforced

Yes	Each CS+ approach was reinforced.
No	None CS+ approach was reinforced.
66%	The first two CS+ approaches were reinforced, but the last was not.
yes+no	Reinforcement was experimentally manipulated (see paper).

Balloon pos

Outside	Balloons were placed at the counter and participants were crossing between table and balloon.
Table	Balloons were placed next to the table and participants must surround them.

Break after pos

0 s	There was no break after the placing of the book in the rack and the next book appeared directly.
2 s	There were 2 seconds pause after one book was put in the rack and before the next one appeared.

Hand-net length

Long	handle length: 0.75 m; net diameter: 0.40 m
Short	handle length: 0.35 m; net diameter: 0.40 m

6 Unity-Scenes

6.1 Installation / Setup

1. Create a new Unity 3D Project
2. Download and import all necessary assets from the asset store (see list below)
3. Import the QHAB.unitypackage

6.2 Required Assets

6.3 For all Scenes

- SteamVR (Version 2.5.0) (**free**)
  https://assetstore.unity.com/packages/tools/integration/steamvr-plugin-32647
  - For VR and the balloons
-

6.3.1 FearConditioning

- Old Office Props Free (version 1.0; Jake Sullivan) (**free**)
  https://assetstore.unity.com/packages/3d/props/interior/old-office-props-free-53735
-

6.3.2 Fishing

- A Medieval House (version 1.1; by Dean Hunt) (**free**)
  https://assetstore.unity.com/packages/3d/a-medieval-house-3238
  - For the wood blanks
- SUIMONO Water System (Version 2.1.9; by Tanuki Digital) (**charged**)
  https://assetstore.unity.com/packages/vfx/shaders/suimono-water-system-4387#releases
  - Used for water
- Bush-Craft Starter Pack (by Artistic Mechanics) (**free**)
  https://assetstore.unity.com/packages/3d/props/tools/bush-craft-starter-pack-56928
  - Used for bucket
- Sea fish - underwater world (by Mixaill) (**charged**)
  https://assetstore.unity.com/packages/3d/characters/animals/sea-fish-underwater-world-63600
  - Used for Fish

6.3.3 Path-Choice / TouchTheEnemy

- QA Office and Security Room (version 1.1; by QAtmo) (**charged**)
  https://assetstore.unity.com/packages/3d/environments/urban/qa-office-and-security-room-114109
  - For inventary
- Big Furniture Pack (version 1.3; by Vertex Studio) (**free**)
  https://assetstore.unity.com/packages/3d/props/furniture/big-furniture-pack-7717#content
  - For the weight to hold the balloons
